# A rare case of purulent meningitis caused by Capnocytophaga canimorsus in the Czech Republic – case report and review of the literature

**DOI:** 10.1186/s12879-020-4760-2

**Published:** 2020-02-03

**Authors:** Petr Prasil, Lenka Ryskova, Stanislav Plisek, Pavel Bostik

**Affiliations:** 1 Department of Infectious Diseases, Charles University School of Medicine and Faculty Hospital, Hradec Kralove, Czech Republic; 2 Department of Clinical Microbiology, Charles University School of Medicine and Faculty Hospital, Hradec Kralove, Czech Republic; 3 Faculty of Military Health Sciences, University of Defense, Trebesska 1575, 50001 Hradec Kralove, Czech Republic

**Keywords:** *Capnocytophaga*, Purulent meningitis, Invasive infection

## Abstract

**Background:**

Invasive infections caused by *Capnocytophaga canimorsus* are rare. Immunocompromised patients, who report being bitten by or having a close contact with an animal, represent a high-risk group for this infection. There are only few dozens of infections by this bacteria manifesting as purulent meningitis reported worldwide. The reported case is a first reported case of purulent meningitis caused by by *Capnocytophaga canimorsus* in Czech Republic with only a limited risk factor history.

**Case presentation:**

The patient, a 74 years old man, was referred to the infectious diseases department of a teaching hospital with clear signs of developing purulent meningitis. His anamnestic data did not show any unusual findings. He was treated for compensated diabetes mellitus type II. The blood cultures were negative and the etiological agent did not grow from the cerebrospinal fluid (CSF) on common media. Eventually, it was identified by detecting pan-bacterial DNA and DNA sequencing. Subsequently, the pathogen was confirmed by anaerobic cultivation from CSF. Only after then the patient recalled being bitten by his German shepherd puppy during play. The patient was successfully treated intravenously by ceftriaxone.

**Conclusions:**

Purulent meningitis caused by *Capnocytophaga spp.* is a rare disease, but it needs to be considered in patients at risk with pre-existing conditions, who report close contact with or being bitten by an animal. It is important to test for this microbe in cases with negative microbiological results for the more common agents.

## Background

*Capnocytophaga canimorsus* (genus *Capnocytophaga*, family *Flavobacteriaceae*) is a fermenting Gram-negative rod, which is commonly present in oral microflora in animals and is predominantly found in the saliva of cats and dogs. Human strains, such as *C. gingivalis, C. sputigena, C. ochracea* and others, are found predominantly in immunocompromised patients in gingival plaque in the periodontal disease. Infections of humans usually occur through a penetrating wound or by a skin abrasion caused by a positive animal, or by a contact of a skin wound with animal saliva [1–3]. There are several invasive forms of the *C. canimorsus* infection, which have been described in humans. The most common one is sepsis followed by purulent meningitis. Other, less frequently observed forms, include infections of the eye, soft tissues and bones, endocarditis, or infections affecting pregnant women, such as those of fetal membranes or gynecological infections [4]. The majority of the invasive infections have been described in immunocompromised patients or those with other serious illnesses. In healthy people these infections are rare and, usually, follow a milder course. Risk factors for the development of a serious disease include asplenism, alcohol abuse, smoking, corticosteroid therapy and hemato-oncological diseases [5].

The first infection of humans by *C. canimorsus* was described in 1976 [6].

Incidence rates of positive identification of *C. canimorsus* in animals depend on the diagnostic technique used and vary widely. Thus, Westwell et al. [7] was able to show 24% of canine samples being positive by cultivation. In the work of Umeda [8], however, when both the PCR and cultivation methods were used, *C. canimorsus* was identified in samples of dental plaque swabs from 69,7% of dogs and 54,8% of cats tested.

The pathogenesis of the disease was investigated in both the in vivo model and in vitro experiments on human macrophages. *C. canimorsus* infects the macrophages and replicates in them, but does not interact with Toll-like receptors and therefore does not induce pro-inflammatory cytokines, such as TNF- α, interleukins or IFN-γ [9]. The bacteria is also resistant to the effects of the complement and polymorphonuclear leucocytes [10]. It has been suggested, that the infection elicits only very weak inflammatory response and the bacteria can escape the immune system [11, 12]. The main invasive factors have not been fully described yet, but the lipopolysaccharide and sialidase are on top of the list [13, 14].

Only few dozens of infections by this bacteria manifesting as purulent meningitis in humans have been reported worldwide and the reported case is the first one reported in Czech Republic, which, contrary to the majority of others, had only a limited risk factor history.

## Case presentation

The 74 years old male patient first reported to his general practitioner (GP) on August 24. For the last 3 days he had experienced headache and stiff neck without any fever, for which he had been taking paracetamol. On the day of his visit he started to feel nauseated and his temperature rose to 39^o^ C. Anamnestically, the patient had been on an ongoing treatment for type II diabetes mellitus with metformin, for arterial hypertension with telmisartan/hydrochlorothiazid, for dislipidemia with atorvastatin and for liver steatosis with sillymarin. He had undergone cholecystectomia, abdominal hernia surgery and partial gastric resection for ulcer disease. He had used to work as an engineer in a factory, but he was retired at that point and lived with his wife and dog in a family house. He had had no travel history for the preceding 2 years, had never smoked, alcohol had used only occasionally and had engaged in sports.

The GP reported the following - the patient was febrile, nauseated, with normal blood pressure and pulse, pulmonary signs without pathological findings and abdominal exam showed no local resistance or pain. However an upper meningeal syndrome was present and thus the patient was immediately sent to the local infectious disease (ID) department.

There, on the admission, the patient was febrile, blood pressure and pulse were stable and Glasgow Coma Scale was 15. His laboratory showed increased inflammatory markers (leucocytosis, CRP of 176 mmol/l). Both aerobic and anaerobic blood cultivation was started. X-rays of lungs and paranasal sinuses were negative. Cerebrospinal fluid (CSF) examination showed 5524 segmented neutrophils/mm^3^, 1568 lymphocytes/mm^3^, glucose 2,98 mmol/l and protein 2,1 g/l. A combination therapy of intravenous dexamethazone (4 mg every 6 h), ceftriaxone (4 g every 24 h) and crystalline solution (500 ml applied over 4 h) was started and the patient was transported to the intensive care unit (ICU) of the ID department of the Faculty Hospital in Hradec Kralove on the same day.

At the admission there the patient was clinically stable and conscious. The neurological examination showed only a mild stiffness of his neck and a slight tremor of fingers. The CT scan of the brain and paranasal sinuses showed no pathology and the Ear-Nose and Throat specialist found no infectious foci in this area. Both aerobic and anaerobic CSF cultivation in blood culture bottles (Bactec 9240, Becton Dickinson) were started. Microscopic and agglutination analysis of the CSF were negative. The therapy with corticosteroids was continued for another 2 days. Intravenous ampicillin was added to the therapy (3 g every 6 h), but only four doses were administered, because Listeria spp. was soon ruled out as the causative agent in the next step (below). Due to the hyperglycemia, insulin was administered intravenously.

As there was no growth detected by the CSF cultivation on the second day, the cultivation analysis was continued further to the total length of 7 days and molecular diagnostic methods were implemented.

During the first step, PCR tests performed on CSF ruled out *Streptococcus pneumoniae, Neisseria meningitidis, Listeria monocytogenes, Staphylococcus aureus* and *Haemophilus influenzae.* The second day the CSF values showed a slight improvement and transoesophageal ultrasound of the heart showed no signs of endocarditis.

**Fig. 1:**
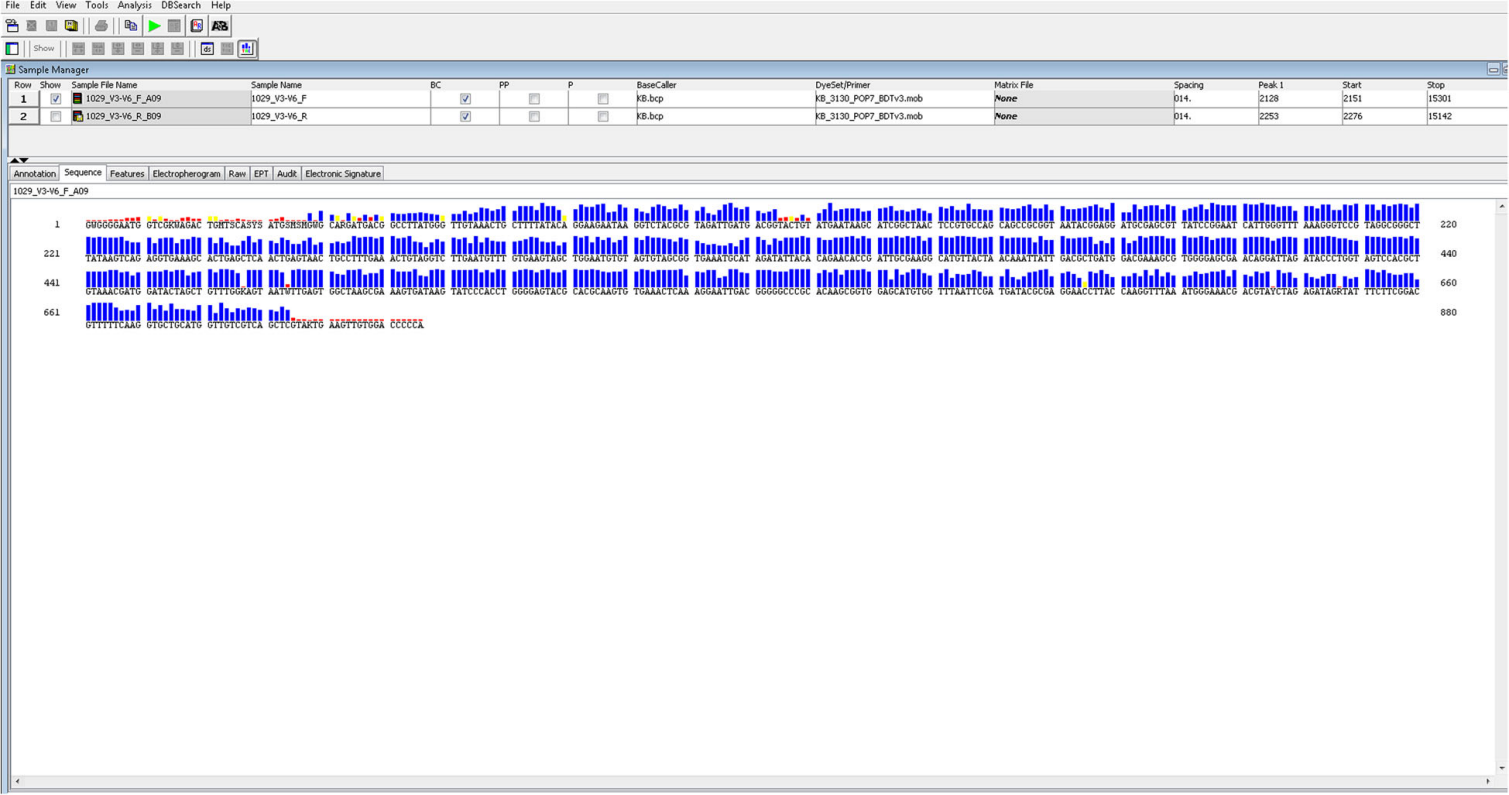
*Capnocytophaga canimorsus* sequence obtained by DNA sequencing from CSF

Due to the still unclear microbial diagnosis vancomycin (1 g in 250 ml intravenously every 12 h) was added to the antibiotic regimen. Since there was a strong PCR positivity for the pan-bacterial DNA from the CSF, targeted PCR for *Escherichia coli* and *Mycobacterium tuberculosis* was performed, but with negative results. However, a subsequent bacterial DNA sequencing on August 28 identified DNA of the *Capnocytophaga spp*. with a specificity of 99% (Fig. 1). No further specification could be achieved.

Simultaneously the anaerobic CSF cultivation using the Schaedler agar (anaerobic chamber with atmosphere containing 85% nitrogen, 5% hydrogen and 10% carbon dioxide, temperature 37 °C, incubated for 7 days total) started to show small semi-transparent colonies. Microscopically they were formed by fusiformic Gram-negative small rods. MALDI-TOF (Bruker Daltonik) identified *Capnocytophaga canimorsus* with a score of 1,98. Blood and other biological samples were negative.

The antibiotic therapy was adjusted to intravenous ceftriaxone (4 g) only. This led to a subsequent improvement of the overall status and to a decrease of inflammatory markers. The antidiabetic therapy was converted to oral administration of metformin (500 mg daily in one dose) and, on September 8, the control exam of the CSF was negative. An examination by a stomatologist ruled out any infectious foci in the respective area.

**Fig. 2:**
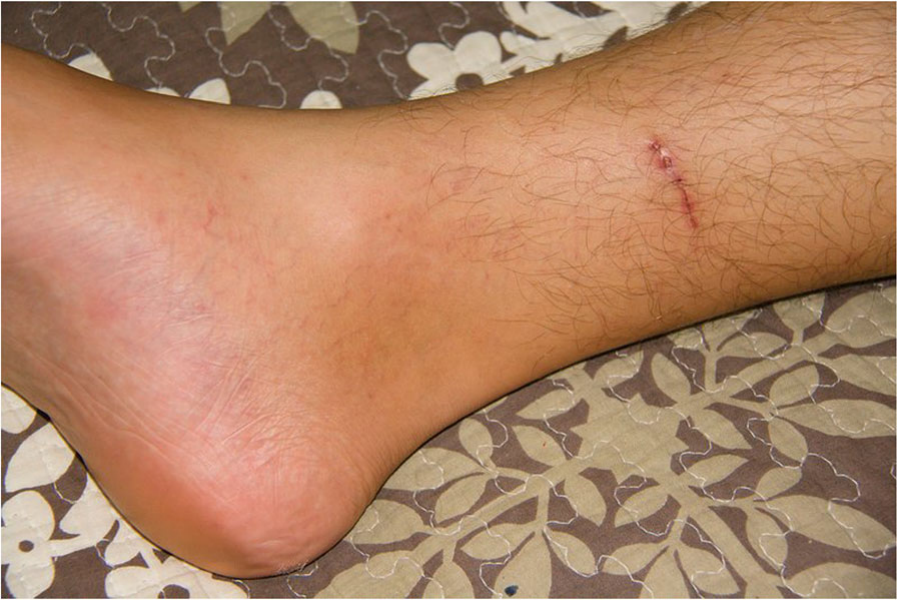
An almost healed small bruise above patient’s ankle inflicted by his puppy of the German shepherd

**Fig. 3:**
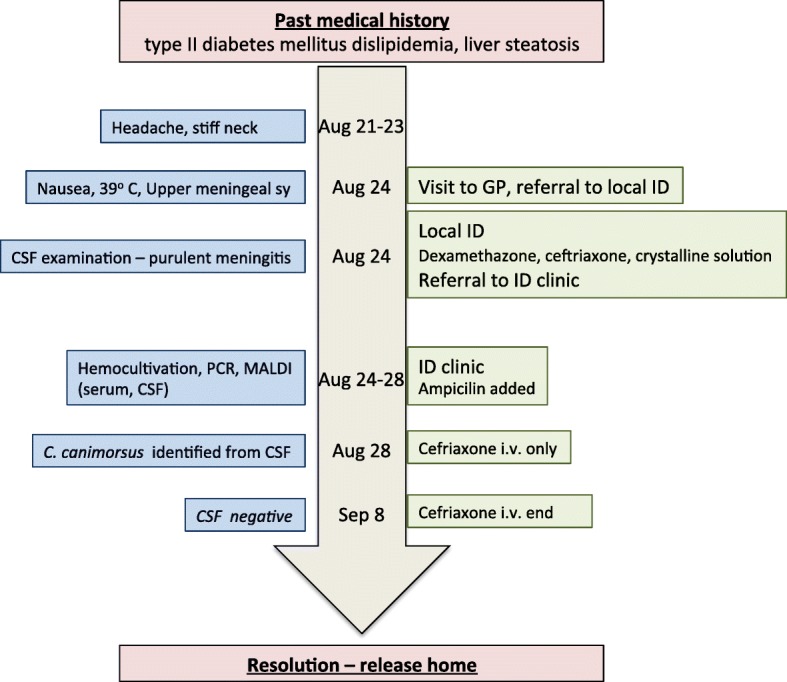
Case timeline

During the subsequent days the patient recalled being bitten by his 4 months old German shepherd dog on the internal side of his right shank approximately 2 weeks before the symptoms started. The wound was not bleeding and was painless. After careful examination the attending physician discovered an almost healed wound about 5 cm long, which was not discovered during any of the physical exams before (Fig. 2). On day 14 after admission the antibiotic treatment was stopped and the patient was released from the hospital on day 15 fully recovered (Timeline in Fig. 3).

## Discussion and conclusions

This is the second reported case of an invasive infection caused by *C. canimorsus* in the Czech Republic (CZ). However, the first one manifested as a severe sepsis in a woman with splenectomy [15]. Thus the patient described in this report is the first case with purulent meningitis reported in CZ. It is possible though, that the numbers of undescribed cases are likely higher in both CZ and worldwide. Butler et al. described a cohort of 484 cases of laboratory confirmed cases of *Capnocytophaga spp*. infections worldwide in 2015 [16]. About two thirds of these patients were men, with the age median of 55 years and mortality in this group was 26%. Clinical symptoms included severe sepsis with septic shock, gangrene, meningitis, endocarditis and eye infections. Majority of all patients reported some contact with animals as a likely source – 60% of them were bitten by a dog and another 27% recalled bruises, scratches, licking or other types of close contacts caused by cats or dogs. Meningitis (33 cases) was described mostly in older patients, often men, with longer incubation periods than in septic cases, and showed mortality rates lower than 5% [16–18]. The data from this cohort thus confirm meningitis as a relatively rare manifestation of this infection (6,8%). However, this prevalence is comparable to the rates observed for purulent meningitis in infections caused by other pathogens – e.g. *Streptococcus pyogenes* - 5,4 to 7,7%, *Streptococcus pneumoniae* and *Haemophilus influenzae* both around 4% [19–23]. On the other hand, the prevalence of purulent meningitis in infections caused by *Neisseria meningitidis* is substantially higher, between 40 and 65%, depending on individual serotypes and regional differences [24–28]. Reasons for lower mortality rates in patients with purulent meningitis (in comparison with sepsis) caused by *Capnocytophaga spp*. are likely similar to those in meningitis caused by meningococci (in comparison with meningococcal sepsis) – the patients are not likely to develop lethal complications of sepsis, such as disseminated intravascular coagulation, thrombocytopenia, Waterhouse-Fridrichsen syndrome and others.

Because the number of cases of invasive infections caused by *Capnocytophaga spp*. is low to begin with, the cases of purulent meningitis are very rare and thus the identification of the causative agent is difficult and complicated, since the CSF is often the only lab accessible compartment positive for this bacteria. In addition, the “classical” laboratory methods are often not reliable – conventional biochemical testing in many cases does not provide diagnostically relevant answers, the bacteria grow slowly and require specific conditions - increased CO_2_, the chocolate agar or selective media with antibiotics [3, 14, 17, 29] . These are all the reasons that lead to false negatives caused mostly by the cultivation of the CSF on just the basic media. Therefore, the molecular methods involving PCR represent the main tool for the successful diagnosis. Even these are not trivial and available in all hospitals, as they require the ability to amplify pan-bacterial DNA and subsequently to identify the culprit by sequencing.

It is not surprising that infections by *Capnocytophaga spp* occur mostly in immunocompromised patients, as the impaired immune response coincides with the ability of this microbe to evade the immune response [9–12]. However, it is “only” 29,5% of the patients with invasive forms of disease, who are immunocompromised in a more severe manner (14,2% with splenectomy or hyposplenism, 12% alcohol abuse, 1,3% hematooncologic diseases, then also HIV infection, diabetes mellitus and corticosteroid therapy) [16]. The patient described in this report was not in any clear immunosuppression. The only other comorbidity was diabetes mellitus type II treated with oral antidiabetic drugs. However, even this disease thus represents a risk factor for the infection by *Capnocytophaga spp*.

Antibiotics of the β-lactam structure represent a mainstream of therapy of these infections. Due to the potential of these bacteria to produce beta-lactamases, however, cephalosporins of higher generations, such as ceftriaxone or cefepime are usually administered parenterally. Amoxicilin, amoxicilin/clavulanate, piperacilin/tazobactam or chloramphenicol also show good therapeutic responses. Carbapenems are indicated for mixed soft-tissue infections or multi-resistant microbial strains. Clindamycine, doxycycline, amoxicilin/clavulanate or fluorochinolones administered orally show good results in less severe infections. On the other hand aminoglycosides, anti-staphylococcal penicillins, colistine and trimethoprim-sulfametoxazole have no therapeutic effects. The sensitivity of *Capnocytophaga spp*. to macrolidic antibiotics, aztreonam, phosphomycine, rifampicine and first-generation cephalosporins is questionable. In the cases of open and bleeding wounds caused by animal bites it is recommended to treat the patient with antibiotics orally and verify their tetanus vaccination history [3, 14, 16, 17, 29, 30]. The patient described in this report was successfully treated by intravenous cefriaxone.

Purulent meningitis caused by *Capnocytophaga spp.* is a rare disease, but it needs to be considered in patients at risk with pre-existing conditions, who report close contact with or being bitten by an animal. It is important to test for this microbe in cases with negative microbiological results for the more common agents. There are hundreds of cases of invasive infections (sepsis) caused by *Capnocytophaga spp.* reported worldwide, but only dozens of cases of purulent meningitis. It is thus likely that the real numbers of cases of these meningitides are much higher and not diagnosed properly.

## Data Availability

N/A

## Abbreviations

CSF: Cerebrospinal fluid
CT: Computer tomography
CZ: Czech Republic
DNA: Deoxyribonucleic acid
GP: General practitioner
ICU: Intensive-care unit
ID: Infectious diseases department
IFN-γ: Interferon γ
MALDI-TOF: Matrix assisted laser desorption/ionization- time-of-flight
PCR: Polymerase chain reaction
TNF-α: Tumor necrosis factor α
